# Intervening upregulated SLC7A5 could mitigate inflammatory mediator by mTOR-P70S6K signal in rheumatoid arthritis synoviocytes

**DOI:** 10.1186/s13075-020-02296-8

**Published:** 2020-08-31

**Authors:** Jing Xu, Congshan Jiang, Yongsong Cai, Yuanxu Guo, Xipeng Wang, Jiaxiang Zhang, Jiawen Xu, Ke Xu, Wenhua Zhu, Si Wang, Fujun Zhang, Manman Geng, Yan Han, Qilan Ning, Peng Xu, Liesu Meng, Shemin Lu

**Affiliations:** 1 Department of Biochemistry and Molecular Biology, School of Basic Medical Sciences, Xi’an Jiaotong University Health Science Center, Xi’an, 710061 Shaanxi People’s Republic of China; 2 Key Laboratory of Environment and Genes Related to Diseases (Xi’an Jiaotong University), Ministry of Education, Xi’an, 710061 Shaanxi People’s Republic of China; 3 Department of Joint Surgery, Xi’an Hong Hui Hospital, Xi’an Jiaotong University Health Science Center, Xi’an, 710061 Shaanxi People’s Republic of China

**Keywords:** SLC7A5, MMPs, mTOR-P70S6K, Fibroblast-like synoviocytes, Rheumatoid arthritis

## Abstract

**Objective:**

The disruption of metabolic events and changes to nutrient and oxygen availability due to sustained inflammation in RA increases the demand of bioenergetic and biosynthetic processes within the damaged tissue. The current study aimed to understand the molecular mechanisms of SLC7A5 (amino acid transporter) in synoviocytes of RA patients.

**Methods:**

Synovial tissues were obtained from OA and RA patients. Fibroblast-like synoviocytes (FLS) were isolated, and SLC7A5 expression was examined by using RT-qPCR, immunofluorescence, and Western blotting. RNAi and antibody blocking treatments were used to knockdown SLC7A5 expression or to block its transporter activities. mTOR activity assay and MMP expression levels were monitored in RA FLS under amino acid deprivation or nutrient-rich conditions.

**Results:**

RA FLS displayed significantly upregulated expression of SLC7A5 compared to OA FLS. Cytokine IL-1β was found to play a crucial role in upregulating SLC7A5 expression via the NF-κB pathway. Intervening SLC7A5 expression with RNAi or blocking its function by monoclonal antibody ameliorated MMP3 and MMP13 protein expression. Conversely, upregulation of SLC7A5 or tryptophan supplementation enhanced mTOR-P70S6K signals which promoted the protein translation of MMP3 and MMP13 in RA FLS.

**Conclusion:**

Activated NF-κB pathway upregulates SLC7A5, which enhances the mTOR-P70S6K activity and MMP3 and MMP13 expression in RA FLS.

## Background

Rheumatoid arthritis (RA) is a chronic autoimmune disease with a global prevalence of 0.24% [1], characterized by synovial hyperplasia and progressive destruction of mainly the small joints. Many cell types, including T cells, B cells, macrophages, and fibroblast-like synoviocytes (FLSs), participate in the complex mechanism of RA pathogenesis. FLSs in the lining of the synovium play a major role and express high levels of inflammatory cytokines that perpetuate inflammation and proteases that degrade the cartilage [2]. Furthermore, RA FLSs are described to present a tumor-like phenotype [3], with increased invasiveness into the extracellular matrix (ECM), which further exacerbates synovial hyperplasia and joint damage [4, 5]. Meanwhile, these quickly proliferated FLS demand high energy, which is well associated with high-level transportation and consumption of glucose and amino acids.

Solute carrier family 7 member 5 (SLC7A5), alias L-type amino acid transporter (LAT1) [6], is a sodium-independent high-affinity amino acid transporter. SLC7A5 together with SLC3A2 mediates cellular uptake of the large neutral amino acids such as phenylalanine, tyrosine, leucine, and tryptophan [7]. The *SLC7A5* is mainly distributed in the placenta, testis, bone marrow, and brain, whereas *SLC3A2* is expressed ubiquitously in all tissues [8]. Global knockout of *Slc7a5* resulted in an embryonic lethal phenotype in mice, and it may be partly due to a deleterious effect upon Slc7a5 transport function during post-implantation embryonic development [9, 10]. The conditional knockout of *Slc7a5* showed that Slc7a5 worked as a checkpoint in T cell activation via the mTORC1 complex [11]. Meanwhile, the hypoxia-inducible factor 2α binds to the *SLC7A5* proximal promoter and drives its transcription in the WT8 cell line [12]. In the inflamed RA joints, the hypoxic condition becomes gradually severe due to increased metabolic demand of the active cells and due to inadequate oxygen delivery through poor perfusion of the inflamed joint [13]. Recently, an mRNA expression profiling study has documented the elevated levels of SLC7A5 in RA synovial tissue [14].

Different studies have indicated the potential role of SLC7A5 in RA pathogenesis; however, much is not known about its actual function in the inflamed FLS. This study was proposed to explore the potential role of SLC7A5 and understand the underlying molecular mechanism in FLS of RA patients.

## Methods

### Patients’ samples

Synovial tissues and FLS were derived from patients with RA and OA who underwent surgical knee joint replacement (Department of Joint Surgery, Honghui Hospital, Xi’an Jiaotong University, China). All the patients’ data are summarized in Table 1. All participants gave their written informed consent prior to inclusion in the study. The study was approved by the Medical Ethics Committee of Xi’an Jiaotong University (No. 2016-261 and No.2017-666).

**Table 1:** Patient characteristics

Clinical data	RA	OA
Number of patients	24	24
Sex		
Female	17	19
Male	7	5
Age^#^	56.71 ± 1.673	66.38 ± 1.396
CRP^#^ (mg/L)	29.30 ± 3.615	4.25 ± 1.608
RF^#^ (IU/mL)	92.57 ± 15.07	6.846 ± 0.9816
ESR^#^ (mm/h)	64.83 ± 7.18	15.79 ± 3.774

*CRP* C-reactive protein, *RF* rheumatoid factor, *ESR* erythrocyte sedimentation rate

^#^Mean ± SEM

### Histology and immunofluorescence

For routine histopathological analysis, paraffin-embedded synovial tissue sections from RA and OA patients were deparaffinized and stained with hematoxylin and eosin (H&E). For immunofluorescence staining, 6-μm-thick tissue sections were incubated overnight at 4 °C with the following primary antibodies diluted in PBS: mouse monoclonal antibody against SLC7A5 (1:100, Santa Cruz, sc-374232) and rabbit polyclonal antibody to vimentin (1:100, Bioss, bs-23064R). Next morning, the samples were washed three times in PBS and incubated for 45 min at room temperature with secondary antibodies, i.e., FITC AffiniPure goat anti-mouse IgG (H+L) (1:400, Earthox, E031210-01) and Cy3 AffiniPure goat anti-rabbit IgG (H+L) (1:400, Earthox, E031620-01). 4′,6-Diamidino-2-phenylindole (DAPI) was used to detect the nucleus (1:100,000, Sigma-Aldrich, D9542). Immunofluorescent staining procedure was followed with slight modifications, as previously described [15]. The immunofluorescent images were captured with a fluorescence microscope (Olympus, Japan) and analyzed by the ImageJ software.

### Cytokines and inhibitor treatment

Cells were treated with IL-1β (20 ng/mL), TNF-α (20 ng/mL), IFN-γ (20 ng/mL), IL-6 (20 ng/mL), and IL-17A (20 ng/mL) (Genscript, China) for 24 h, and total protein analysis was performed using Western blotting assay.

The samples were incubated with JNK inhibitor SP600125 (10 μM, Selleckchem, s1460), NF-κB inhibitor BAY_11-7085_ (10 μM, Selleckchem, s7352), or P38 inhibitor SB203580 (10 μM, MEC, HY10256A) for 4 h, followed by the addition of 20 ng/mL IL-1β for 24 h, to stimulate the cells. The expression at the mRNA and protein levels was determined by RT-qPCR and Western blotting, respectively.

### Blocking assay of SLC7A5

SLC7A5 antibody (20 μg/mL, a mouse anti-SLC7A5 monoclonal antibody, IgG_1_, Santa Cruz, USA) was administrated to the FLS, following the procedure as detailed in our previous paper [16]. Briefly, FLSs were seeded in 12-well plates at a density of 4 × 10^4^/mL and incubated with SLC7A5 antibody or isotype-matched IgG_1_ (CST, #5415, USA) for 24 h. The cells were then treated with IL-1β for 18 h and collected to detect the mRNA and protein levels of MMP3 and MMP13.

### Western blotting

Total protein lysates from synovial tissues and cells were extracted by using the RIPA solution (Beyotime, China) with a cocktail of protease and phosphatase inhibitors (Roche). The total protein concentration of each sample was determined by a BCA Protein Assay kit (Thermo Scientific, USA). Subsequently, 20 μg from cell lysates was separated by 6% or 8% SDS-PAGE gels and transferred to the polyvinylidene fluoride membrane (EMD Millipore, Billerica, MA, USA). The membrane was incubated with primary antibodies at 4 °C overnight. The list of primary antibodies is depicted in supplemental Table S5. After washing, the membrane was further incubated with a horseradish peroxidase-conjugated goat anti-rabbit or goat anti-mouse IgG secondary antibody (0.4 μg/mL, Abcam, USA) for 2 h at room temperature. Signal intensity was determined by the Supersignal® West Pico Kit (Thermo Scientific) using the enhanced chemiluminescence detection system (EMD Millipore). The band density was measured by the ImageJ software normalized to β-actin.

### RNA isolation and RT-qPCR

Total RNA from the synovial tissues and cells was isolated using the TRI Reagent™ solution (Thermo Scientific, USA) and reverse transcribed to cDNA using the First Strand cDNA Synthesis Kit (Thermo Scientific, USA) according to the manufacturer’s instructions. RT-qPCR was performed by using the iQ5 optical system software (Bio-Rad Laboratories, USA) with Fast Start Universal SYBR Green Master (ROX) (Roche, USA) for relative quantification of the target genes at mRNA level. Gene expression analyses were calculated by 2^−ΔΔCt^ method.

### RNAi

Small interfering RNAs (siRNAs) targeting SLC7A5 (si1: 5′-CATTATACAGCGGCCTCTTT-3′, si2: 5′-TAGATCCCAACTTCTCATTT-3′) and the negative control (NC, 5′-GCGACGAUCUGCCUAAGAUTT-3′) were purchased from Oligobio (Beijing, China). Cells were transfected with 75 nmol/L of either SLC7A5 siRNA or NC siRNA using Lipofectamine™ 2000 Transfection Reagent (Thermo Scientific, USA) according to the manufacturer’s guidelines. The cells were collected for RNA or protein isolation 24–48 h post-transfection, where indicated to detect the treatment effects and the signal pathways.

### Cytokine profiling assay

RA FLSs were seeded in 6-well plates (2 × 10^5^ cells/mL) and incubated overnight in DMEM medium containing 5% FBS. Subsequently, the cells were transfected with siRNA, and 4 h later, the medium was replaced by containing 0.2% FBS and incubated for 48 h. Supernatants were collected and centrifuged (at 2000 rpm for 10 min at 4 °C), and aliquots were stored at − 80 °C before further analyses.

Cytokine expression in siRNA-treated RA FLS supernatants was detected by using RayBio® C-Series human cytokine antibody array (AAH-CYT-5). Dot ELISA-based membrane coated with 80 human cytokines (listed in supplemental Table S2) was incubated with RA FLS supernatants pooled from 4 donors, transfected with si-*SLC7A5* or si-*NC* for 48 h. The detection and analysis of the cytokine array were performed by RayBiotech Company according to the manufacturer’s instructions. Dot immunoblot signals from the membrane array were captured, and the raw intensity was calculated as shown in supplemental Table S3.

### Amino acid deficiency and supplement assay

Lab self-made DMEM were followed by Dulbecco’s modified Eagle’s medium (DME) formulation recipe in the Sigma-Aldrich website. The single amino acid-deficient medium was prepared at the laboratory based on the Dulbecco’s modified Eagle’s medium (DMEM) formulation from Sigma-Aldrich lacking either phenylalanine (Phe) or tryptophan (Trp). For the amino acid supplement assay, additional 1 mM phenylalanine (Phe), tryptophan (Trp), or kynurenine (Kyn) were added into the DMEM medium. The FLSs were cultured in a single amino acid deficiency medium initially for 8 h before the addition of IL-1β into the treatment group medium and incubated for another 16 h. The cells were collected for mTOR activity and MMP expression analyses.

### Statistics

Data were expressed as mean ± standard error of mean and SPSS software was used for statistical analyses. One-way ANOVA among the groups and Student’s *t* test or Mann-Whitney-Wilcoxon test between the two groups were used to determine significant differences according to the distribution of the data (normal distribution was validated using Shapiro-Wilk test). *p* less than 0.05 was considered statistically significant.

## Results

### SLC7A5 expression is upregulated in fibroblast-like synoviocytes from RA patients

**Fig. 1:**
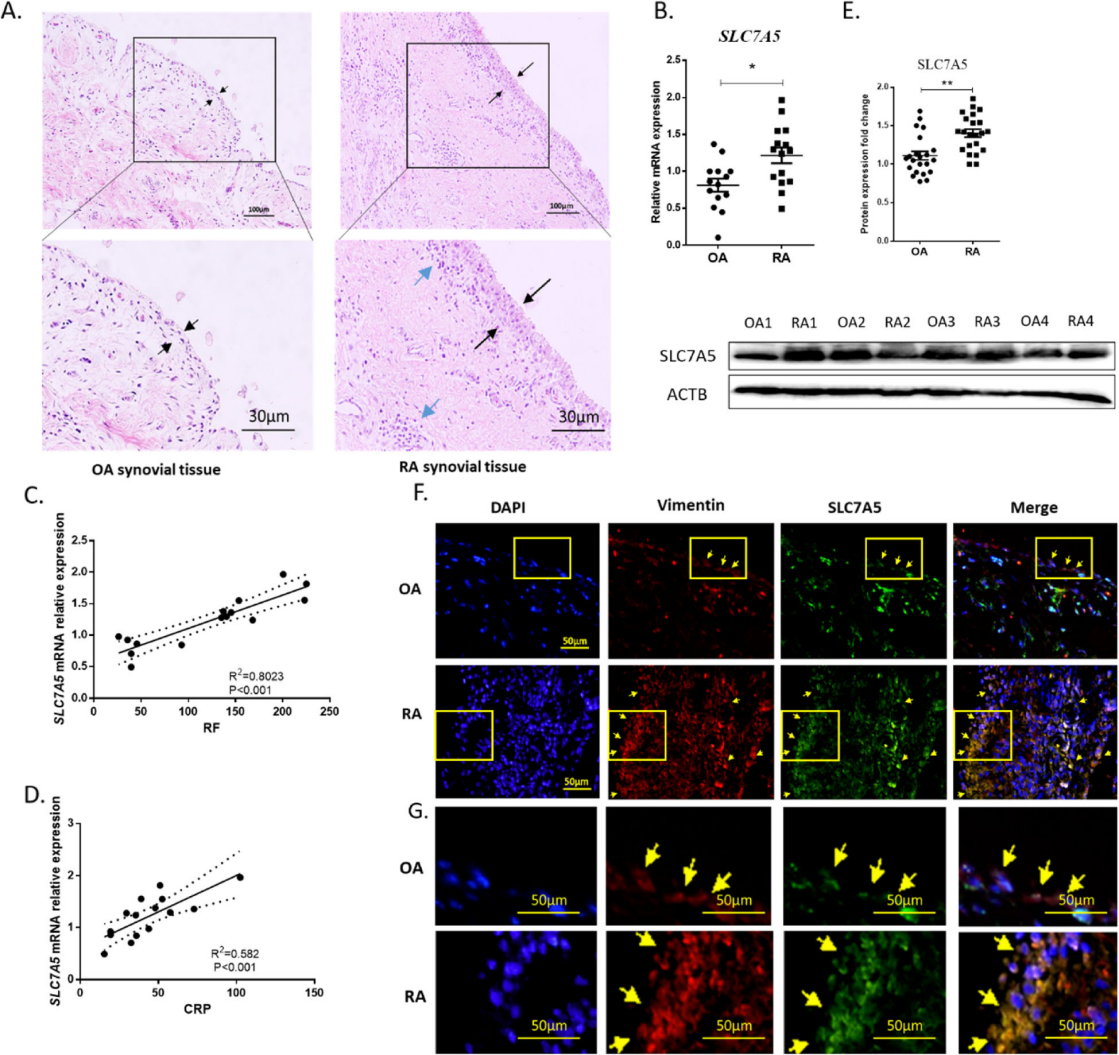
SLC7A5 expression in fibroblast-like synoviocytes of RA patients. **a** Hematoxylin and eosin (H&E) staining of the synovial tissue from RA and OA patients. Black arrows represent the lining cells of synovial tissues, the blue arrows represent the inflamed cells in synovial tissues. **b** The mRNA expression of *SLC7A5* in synovial tissue from OA and RA patients, detected by RT-qPCR (RA *n* = 15, OA *n* = 14). Correlation analysis of *SLC7A5* mRNA expression in FLS from RA patients (*n* = 15) with RF (**c**) and CRP (**d**). **e** The protein expression of SLC7A5 in synovial tissues from OA and RA patients detected by Western blotting. The density of SLC7A5 immune-reactive bands was analyzed by using ACTB expression as a loading control (RA *n* = 22, OA n = 22). **f** Representative immunofluorescence staining for SLC7A5 (green) and vimentin (red) in synovial tissue from OA and RA patients (RA *n* = 3, OA n = 3). The slide used for IF stain was consecutively followed slide stained with H&E. The picture shown in **f** was the enlarging arrow area pointed out in **a**. The yellow boxes were amplified in **g**, yellow arrow pointed out the representative staining cells (*p < 0.05)

To investigate the involvement of SLC7A5 in RA pathogenesis, synovial tissues were collected from RA and OA patients. Histological examination revealed that the synovial tissues from RA patients were heavily proliferated and more infiltrated with inflammatory cells (blue arrow), compared with those from OA patients (Fig. 1a). The expression of *SLC7A5* at the mRNA level was significantly upregulated in synovial tissues from RA patients as compared to those from OA patients (Fig. 1b). In addition, we observed a significant positive correlation of *SLC7A5* expression at the mRNA level with both RF (Fig. 1c) and CRP (Fig. 1d). Likewise, SLC7A5 expression at the protein level was also found significantly upregulated in synovial tissues from RA patients as compared to those from OA patients (Fig. 1e, Supplementary Fig. S1). Immunofluorescence staining of the synovial tissues from RA patients revealed that SLC7A5 was overexpressed and co-localized in vimentin-positive cells (FLS) (Fig. 1f, g).

### The upregulation of SLC7A5is mediated by IL-1β via the NF-κB pathway

**Fig. 2:**
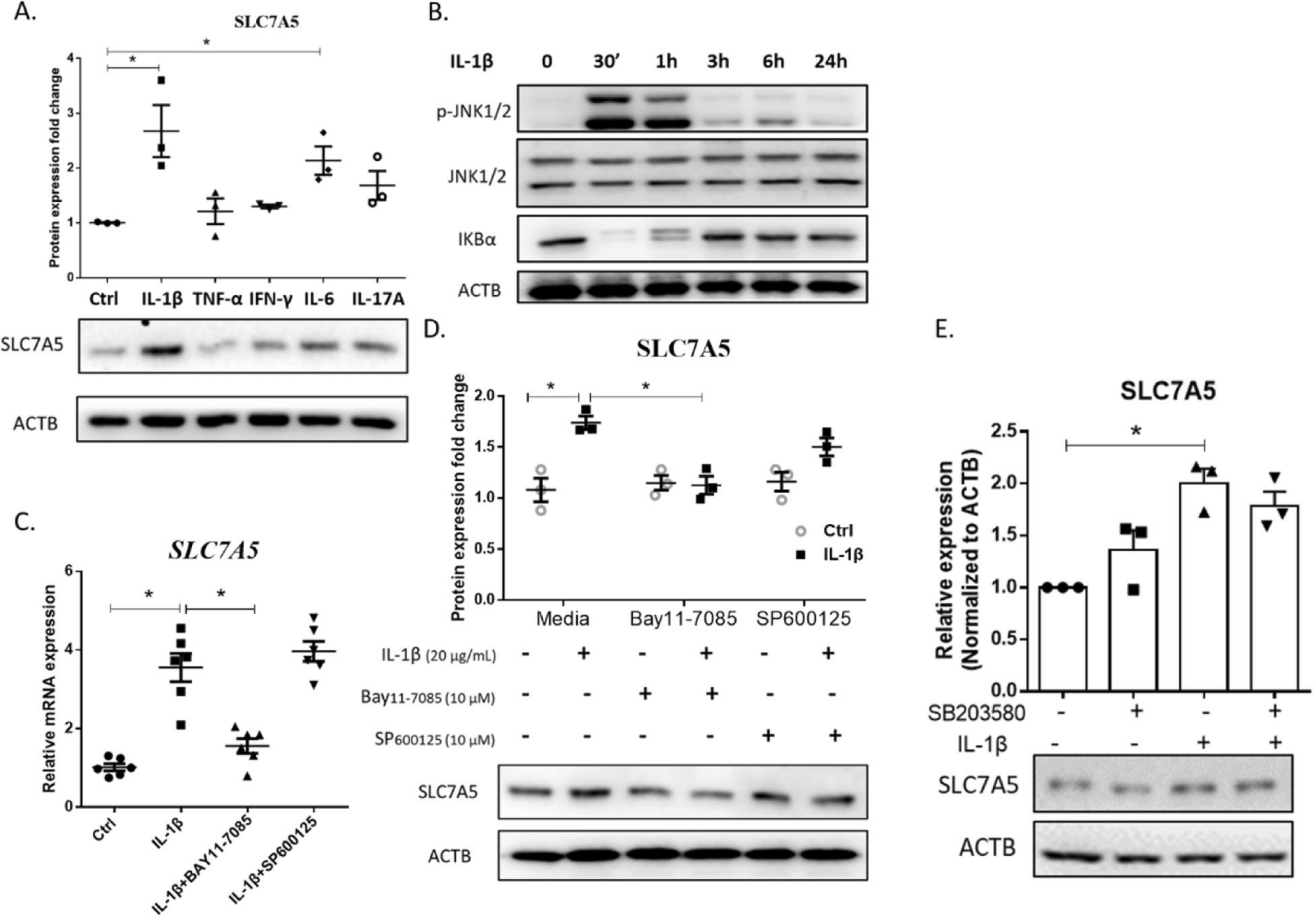
Mechanism of SLC7A5 upregulation in fibroblast-like synoviocytes (FLS) under IL-1β treatment. **a** The protein expression of SLC7A5 in FLS from RA patients treated with different cytokines (IL-1β 20 ng/mL, TNF-α 10 ng/mL, IFN-γ 20 ng/mL, IL-6 20 ng/mL, and IL-17A 10 ng/mL) for 24 h, detected by Western blotting. The density of SLC7A5 immune-reactive bands was analyzed by using ACTB expression as a loading control (*n* = 3). **b** The representative active signaling pathways in FLS from RA patients, detected by Western blotting. The cells were treated with 20 ng/mL IL-1β and collected at different time points for protein isolation. **c** The mRNA levels of *SLC7A5* in FLS from RA patients incubated with different inhibitors and stimulated with IL-1β. The cells were firstly treated with NF-κB inhibitor Bay_11-7085_ (10 μM) or JNK inhibitor SP600125 (10 μM) for 4 h and then treated with 20 ng/mL IL-1β for 24 h. The mRNA expression was detected by RT-qPCR (*n* = 6). **d** The protein expression of SLC7A5 in FLS from RA patients incubated with different inhibitors and stimulated with IL-1β. The cells were firstly treated with NF-κB inhibitor Bay_11-7085_ (10 μM) or JNK inhibitor SP600125 (10 μM) for 4 h and then treated with 20 ng/mL IL-1β for 24 h. The protein expression was detected by Western blotting. The SLC7A5 immune-reactive band density was analyzed by using ACTB expression as a loading control (*n* = 3). **e** The protein expression of SLC7A5 in FLS from RA patients incubated with p38 inhibitor and stimulated with IL-1β. The cells were firstly treated with p38 inhibitor SB203580 (10 μM) for 4 h and then treated with 20 ng/mL IL-1β for 24 h. The protein expression was detected by Western blotting. SLC7A5 immune-reactive bands density was analyzed by using ACTB expression as a loading control (*n* = 3) (*p < 0.05)

To scrutinize which molecule is responsible for the upregulation of SLC7A5 in FLS, we focused on proinflammatory cytokines, the chief sponsors of inflammation in RA. Interestingly, we found that both IL-1β and IL-6 could significantly upregulate SLC7A5 expression at the protein level (Fig. 2a). We used IL-1β to activate both the JNK and NF-κB signaling pathways (Fig. 2b) in FLS, either by phosphorylating JNK or promoting IκB degradation. To know the underlying pathway involved in upregulating SLC7A5 expression, the cells were treated with SP600125 (JNK signaling inhibitor) or Bay_11-7085_ (NF-κB signaling inhibitor) and stimulated by IL-1β. The data revealed that the activated NF-κB signaling played a fundamental role in SLC7A5 upregulation (Fig. 2c, d, Supplementary Fig. S3). We also used SB203580 (P38 pathway inhibitor) in IL-1β-stimulated cells; however, the data showed no involvement of P38 signaling in SLC7A5 upregulation (Fig. 2e).

**Fig. 3:**
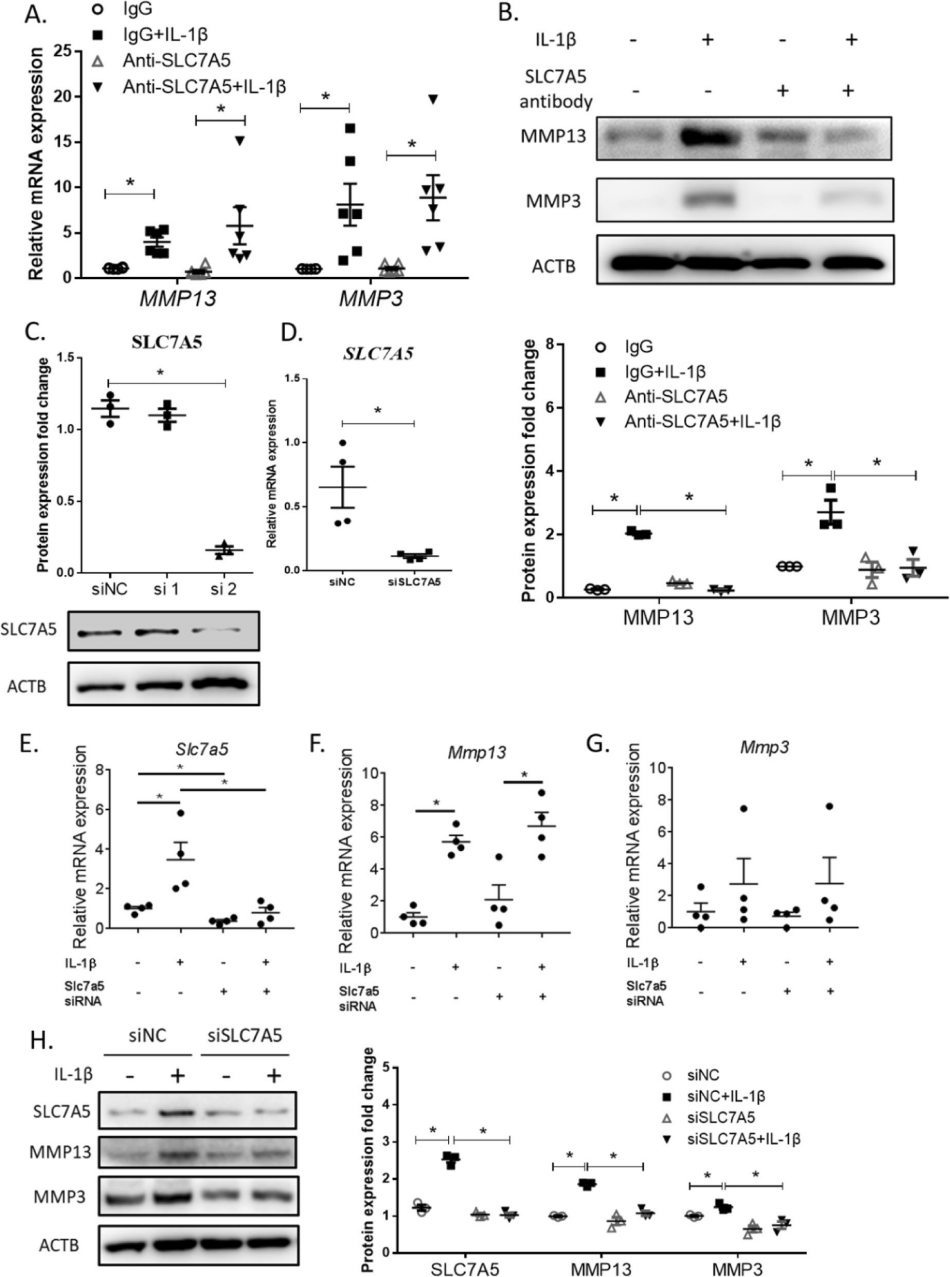
Impact of SLC7A5 intervention either by antibody blocking or siRNA knockdown on the expression levels of MMP3 and MMP13 in FLS from RA patients. **a** The mRNA expression of *MMP13* and *MMP3* in RA FLS, incubated with SLC7A5 monoclonal antibody or isotype IgG and stimulated with or without IL-1β. The cells were incubated with SLC7A5 monoclonal antibody or isotype IgG for 4 h and then treated with or without IL-1β (20 ng/mL) for 8 h. The mRNA levels were measured by RT-qPCR (*n* = 6). **b** The protein expression of MMP13 and MMP3 in RA FLS, incubated with SLC7A5 monoclonal antibody or isotype IgG and stimulated with or without IL-1β. The cells were incubated with SLC7A5 monoclonal antibody or isotype IgG 4 h and then treated with or without IL-1β (20 ng/mL) for 24 h. The protein levels were detected by Western blotting. The density of MMP13 and MMP3 immune-reactive bands was analyzed by using ACTB expression as a loading control (*n* = 3). **c**, **d** Optimization of the SLC7A5 RNAi efficiency in FLS from RA patients. The cells were transfected with siRNA (NC, siSLC7A5-1 or 2) for 24 h, and the protein and mRNA expression levels were detected by Western blotting (**c**) and RT-qPCR (**d**), respectively. **e**–**g** The expression of *SLC7A5*, *MMP13*, and *MMP3* in FLS from RA patients transfected with siRNA (NC or siSLC7A5-2) and treated with or without IL-1β, detected at the mRNA level by RT-qPCR. The cells were first transfected with siRNA (NC or siSLC7A5-2) for 24 h and then treated with or without IL-1β (20 ng/mL) for 8 h without changing the culture medium. **h** The protein expression of SLC7A5, MMP13, and MMP3 in FLS from RA patients transfected with siRNA (NC or siSLC7A5-2) and treated with or without IL-1β. The cells were first transfected with siRNA (NC or siSLC7A5-2) for 24 h and then treated with or without IL-1β (20 ng/mL) for 24 h further, without changing the culture medium. The density of MMP13 and MMP3 immune-reactive bands was analyzed by using ACTB expression as a loading control (*n* = 3) (*p < 0.05)

### Upregulated SLC7A5 enhances MMP3 and MMP13 protein expression in FLS

To figure out the function of SLC7A5 as an amino acid transporter in activated FLS, the SLC7A5 monoclonal antibody was used as a blocker. RT-qPCR results showed that there was no change in the *MMP3* and *MMP13* expression at the mRNA level (Fig. 3a). However, the protein levels of MMP3 and MMP13 were decreased by the SLC7A5 blocker antibody (Fig. 3b, Supplementary Fig. S4A), indicating that the suppression of MMP3 and MMP13 happened only at the protein level.

Two sequences of small interfering RNAs specific to SLC7A5 were synthesized and optimized. siRNA No.2 was found to downregulate the expression of SLC7A5 significantly in FLS 48 h post-transfection both at the protein (Fig. 3c) and mRNA (Fig. 3d) levels. Although the siRNA downregulated the *SLC7A5* expression at the mRNA level successfully, there was no change in the mRNA levels of *MMP3* and *MMP13* (Fig. 3e–g). However, the protein levels of MMP3 and MMP13 were found downregulated by the SLC7A5 siRNA (Fig. 3h, Supplementary Fig. S4B). These results uncovered the involvement of SLC7A5 in regulating MMP3 and MMP13 proteins in RA FLS.

**Fig. 4:**
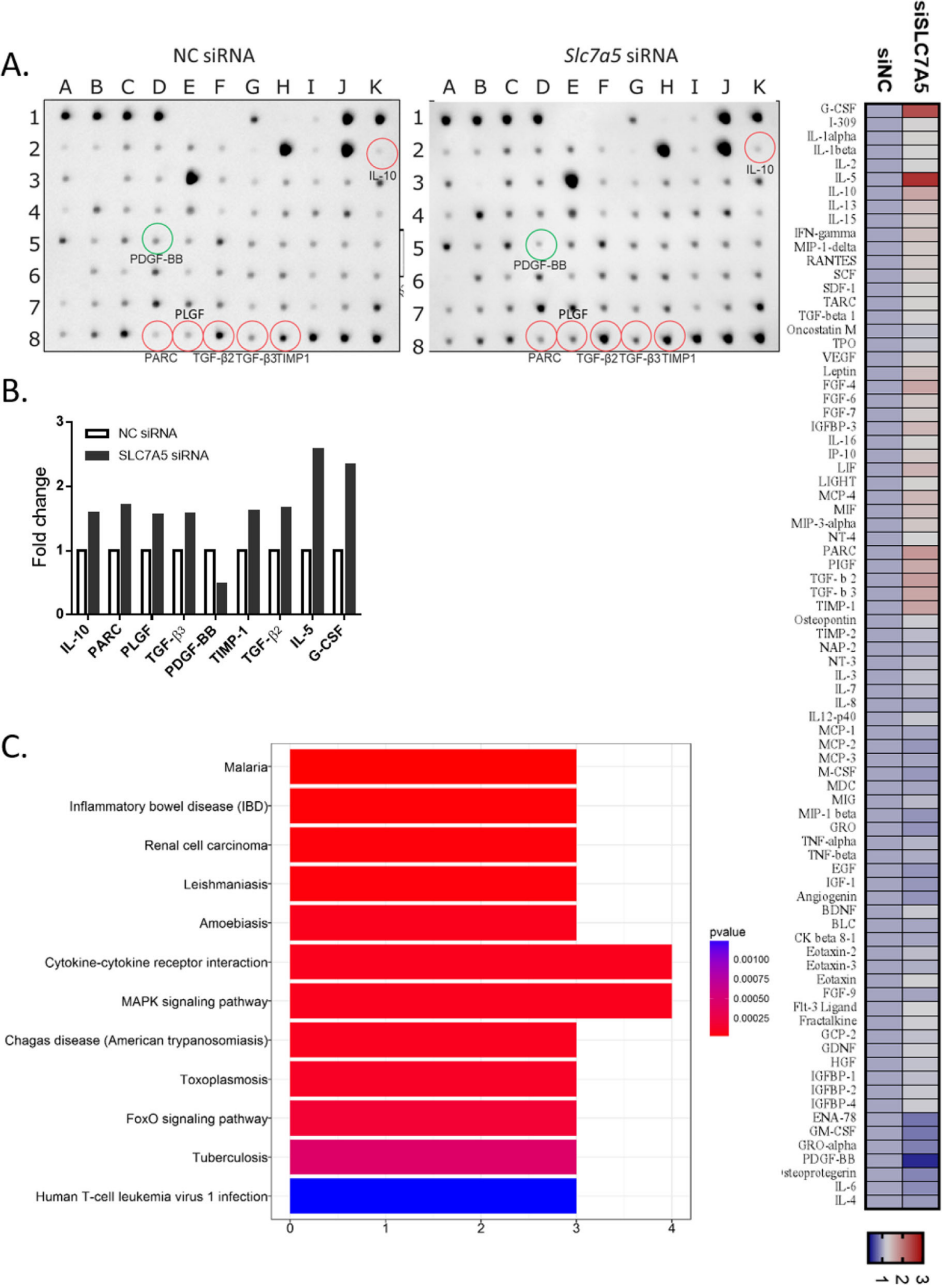
SLC7A5 impact on the production of cytokines and chemokines in RA FLS. **a** Images of cytokine array membranes incubated with supernatants from RA FLS, transfected with NC or SLC7A5 siRNA (si-2). The cells were transfected with either negative control or SLC7A5 siRNA (si-2) for 48 h. All the cytokine and chemokine fold change is shown in the heat map. **b** Semi-quantitative data showing altered cytokine expression (fold change beyond ± 1.5) in RA FLS supernatants 48 h post-siRNA transfection. **c** KEGG pathway analysis of the differentially expressed cytokines and chemokines in RA FLS

A total of 80 human cytokines were detected (Fig. 4a) in conditioned media of RA FLSs transfected with SLC7A5 siRNA for 48 h (Fig. 3c). Semi-quantitative data showed that the fold change increase in the expression of IL-10, PARC, PLGF, TGFβ_2_, TGFβ_3_, and TIMP1 and the fold change decrease in PDGF-BB were beyond ± 1.5 (plotted in Fig. 4b; data shown in supplemental Tables S2, S3 and Supplemental Fig.S2). KEGG pathway analysis predicted that multiple pathways were significantly related to this altered cytokine profiling after *SLC7A*5 knockdown (supplemental Table S4). Among them, it is of particular interest that these pathways also included inflammatory bowel disease (IBD) and rheumatoid arthritis (Fig. 4c).

### Upregulated SLC7A5 activates mTOR-P70S6K signaling and enhances MMP3 and MMP13 expression in FLS

**Fig. 5:**
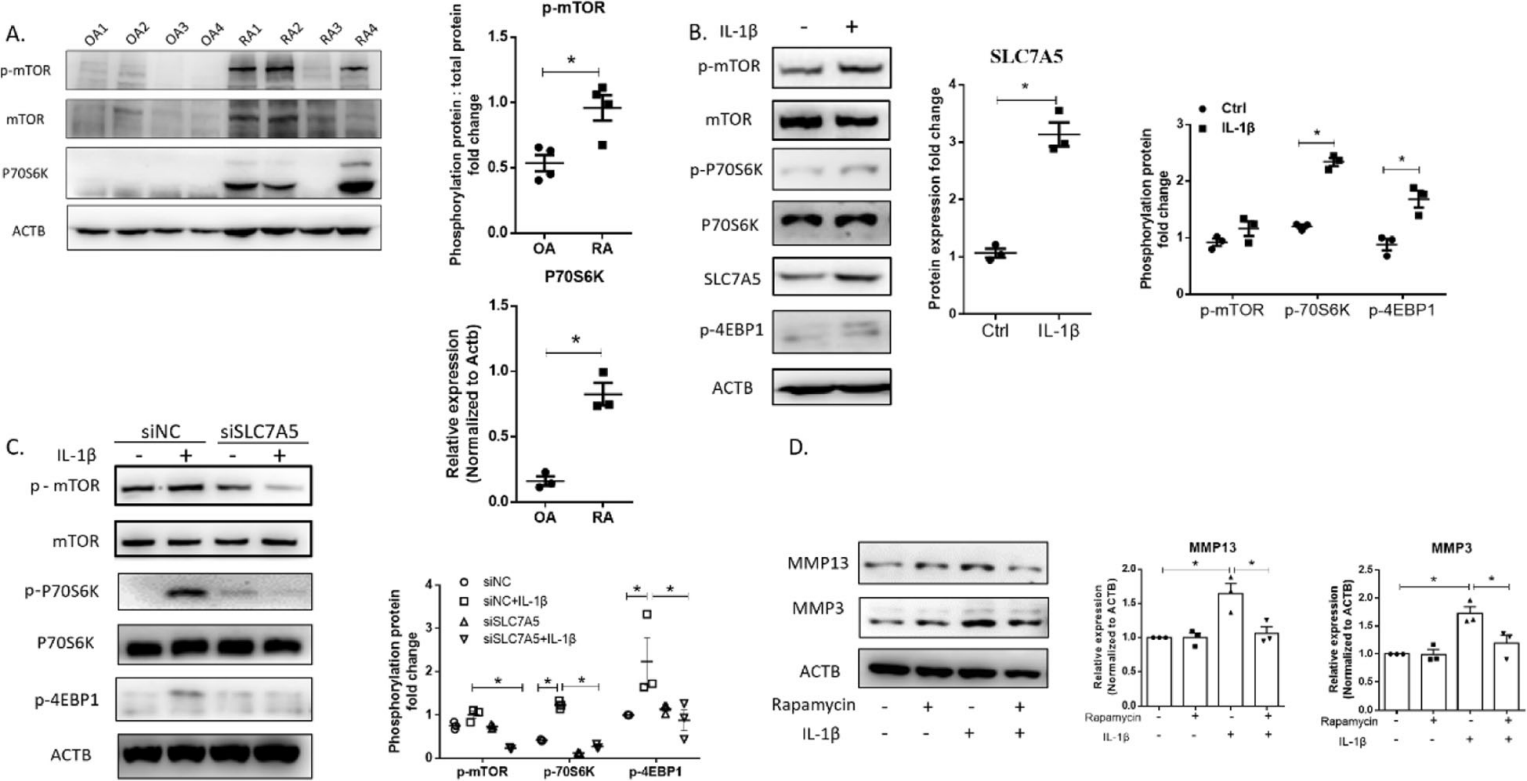
Activation of mTOR-P70S6K signaling and downstream upregulation of MMP3 and MMP13 expression by SLC7A5 overexpressed in RA FLS. **a** The protein expression of p-mTOR (*n* = 4), mTOR, and P70S6K (*n* = 3) in synovial tissue from OA and RA patients detected by Western blotting. **b** The protein synthesis pathway activation in FLS. The FLS were treated with 20 ng/mL IL-1β for 4 h and collected for protein isolation, detected by Western blotting. The density of SLC7A5 immune-reactive bands was analyzed by using ACTB expression as a loading control. The phosphorylation protein ratio fold change of mTOR and P70S6K was analyzed by using total protein expression of their own as a control, while the phosphorylation protein ratio fold change of 4EBP1 was analyzed by using ACTB expression as a loading control (*n* = 3). **c** The impact of SLC7A5 siRNA on the protein synthesis pathway (mTOR-P70S6K-4EBP1) activation in FLS, detected by Western blotting. The cells were transfected with siNC or siSLC7A5 (si-2) for 24 h and then stimulated with 20 ng/mL IL-1β for another 4 h. The fold change in phosphorylated/non-phosphorylated protein ratios of mTOR and P70S6K was analyzed by using total protein expression of their own as a control, while that of 4EBP1 was analyzed by using ACTB expression as a loading control (*n* = 3). **d** The inhibition of MMP3 and MMP13 expression by rapamycin (mTORC1 inhibitor) in RA FLS under IL-1β treatment. The cells were incubated with rapamycin (100 nM) for 8 h and then stimulated with 20 ng/mL IL-1β for another 24 h. The protein levels were detected by Western blotting. The density of MMP3 and MMP13 immune-reactive bands was analyzed by using ACTB expression as a loading control (*n* = 3) (*p < 0.05)

To reveal the mechanism underlying MMP3 and MMP13 regulation at the protein level by SLC7A5, we detected amino acid sensor mTOR and its substrate in synovial tissues and inflamed FLSs. As shown in Fig. 5a, the expression of P70S6K and p-mTOR was significantly upregulated in RA synovial tissues compared with that of OA synovial tissues. IL-1β treatment led to the increased expression of SLC7A5 in FLS accompanied by P70S6K and 4EBP1 phosphorylation (Fig. 5b, Supplementary Fig. S5A). To confirm these results associated with the SLC7A5 role in amino acid sensor activation and signaling pathways involved in the regulation of translation, SLC7A5 was knocked down by RNAi in FLS. We found that phosphorylation of mTOR, P70S6K, and 4EBP1 was significantly intervened by si-SLC7A5 in IL-1β treatment groups (Fig. 5c, Supplementary Fig. S5B). By using mTOR complex 1 (mTORC1) signal inhibitor rapamycin, protein levels of both MMP3 and MMP13 were also decreased significantly (Fig. 5d, Supplementary Fig. S5C). These findings suggest that the overexpressed SLC7A5 in FLS from RA patients has a crucial role in the activation of the mTORC1 pathway and subsequent regulation of the mRNA translation.

### Tryptophan stimulates mTOR activity and enhances MMP3 and MMP13 expression in RA FLS

**Fig. 6:**
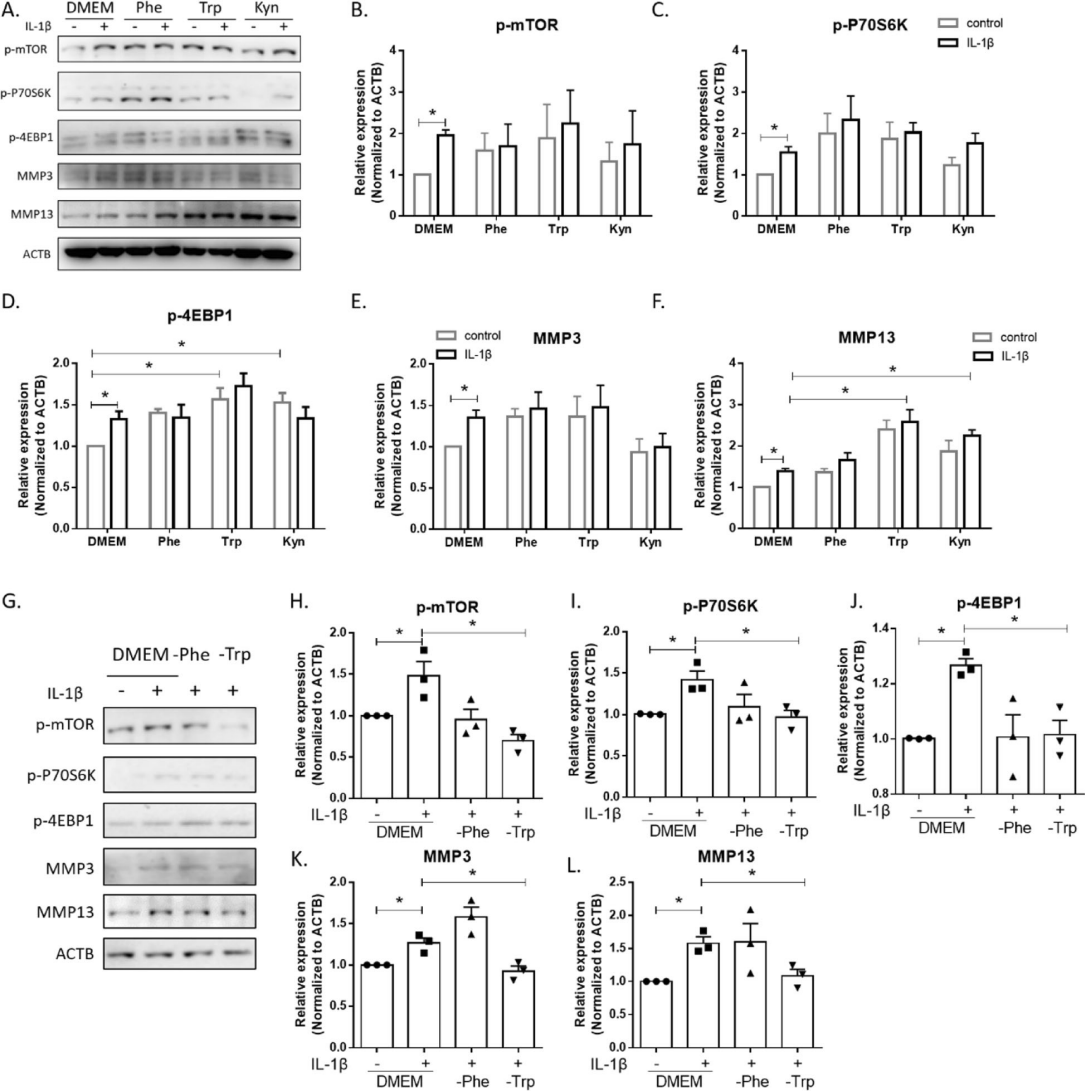
mTOR-P70S6K-4EBP1 activation and downstream upregulation of MMP13 by Trp in FLS from RA patients. FLS were cultured with or without extra Phe, Trp, and Kyn at the concentration of 1 mM for 4 h and stimulated with IL-1β for 24 h. **a** The protein expression levels of p-mTOR, p-P70S6K, p-4EBP1, MMP3, and MMP13 in RA FLS were detected by Western blotting (*n* = 3). mTORC1 activity was assessed by the levels of phosphorylated mTOR (**b**), P70S6K (**c**), and 4EBP1 (**d**), relative to ACTB loading control. The expression of MMP3 (**e**) and MMP13 (**f**) was assessed relative to ACTB loading control. **g** The protein expression levels of p-mTOR, p-P70S6K, p-4EBP1, MMP3, and MMP13 in RA FLS were detected by Western blotting (*n* = 3). The cells were cultured with or without Phe and Trp in DEME for 24 h and then stimulated with IL-1β for another 8 h. mTORC1 activity was assessed by the levels of phosphorylated mTOR (**h**), P70S6K (**i**), and 4EBP1 (**j**), relative to ACTB loading control. The expression levels of MMP3 (**k**) and MMP13 (**l**) were assessed relative to ACTB loading control

Downregulation of SLC7A5 (either by siRNA or through antibody blocking) led to the decreased expression of MMP3 and MMP13 via inhibition of mTORC1 signaling in IL-1β treated FLS. We speculated the possible role of the amino acid transported via SLC7A5 in regulating MMP3 and MMP13 expression via mTORC1 signaling. To check that, extra amino acids (Phe, Trp, or Trp metabolic product Kyn) were added into the culture medium. As shown in Fig. 6a–f, only MMP13 could be upregulated significantly in the IL-1β treatment group after the addition of extra Trp or Kyn (Supplementary Fig. S6). Meanwhile, the addition of extra Trp or Kyn could active the mTOR complex 1 signaling, as measured by 4EBP1 phosphorylation (Fig. 6d). For further investigations, FLSs were cultured in single amino acid (Phe or Trp)-deficient medium. We observed that the expression levels of both MMP3 and MMP13 were decreased significantly in the IL-1β treatment groups, under Trp deficiency (Fig. 6k, l), along with the reduction of the phosphorylated mTOR, P70S6K, and 4EBP1 (Fig. 6h–j). All these data suggest that the upregulated SLC7A5 may transport more special amino acid like Trp and regulate the MMP3 and MMP13 protein expression.

## Discussion

In the present study, we demonstrated that the highly expressed SLC7A5 in the synoviocytes is induced by proinflammatory IL-1β via NF-κB signaling activation. Overexpressed SLC7A5 promotes mTOR-P70S6K signals and enhances the expression of MMP3 and MMP13 at the protein level in RA FLS.

Studies regarding the extent of metabolic changes and the types of metabolites involved may provide us suitable biomarkers for RA diagnosis. Mounting evidence supports the notion that the metabolic changes occurring in the pathogenesis of RA are different from those found in other arthritis diseases [17]. Multiple amino acids such as glycine, leucine, serine, tyrosine, isoleucine, and proline have been reported in the synovial fluid of patients with RA [18]. However, only a few studies are available focusing on the involvement of amino acid transporter in RA pathogenesis. The metabolic changes in joint inflammation are complicated, and many interactions may take place, leading to a complex communication network between different cell types. Therefore, more knowledge is needed to unveil the critical interactions between amino acid transporter and FLS function in the arthritis process. In this study, we established that an amino acid transporter, SLC7A5, is overexpressed in FLS of RA patients, resulting in the upregulation of the MMPs at the protein level, which plays a critical role in maintaining FLS invasive phenotype and degradation of the extracellular matrix. Blocking SLC7A5 activity may slow down the FLS infiltration hence reducing MMP3 and MMP13 expression in RA development.

To understand the mechanism of SLC7A5 upregulation in RA FLS, the FLS inflammatory microenvironment was studied in this study. In RA synovial fluid, a lot of inflammatory mediators are secreted by immune cells. IFN-γ and TNF-α are secreted by activated T cells [19]. IL-17 is produced by Th17 and mast cells [20]. Activated macrophages have been reported to secrete other cytokines like IL-1β, IL-6, and TNF-α [21]. These inflammatory cytokines are well known to accelerate the process of matrix degradation in RA. Hence, we used a series of cytokines to stimulate FLS and observed the behavior of SLC7A5 in vitro. We found that IL-1β-treated cells exhibited upregulated *SLC7A5* expression via NF-κB activation. Yoon et al. have recently demonstrated that SLC7A5 expression was upregulated by LPS in RA monocytes [22]. More interestingly, HIF-2α was found binding to the *Slc7a5* promoter and increased the Slc7a5 expression in normal liver and lung tissues [12]. Hypoxia is an important micro-environmental characteristic of RA, and hypoxia-inducible factors (HIFs) are key transcriptional factors that are highly expressed in RA synovium and are reported to modulate the expression of mediators that are involved in cellular infiltration of the synovial tissue, cartilage destruction, and bone erosion [23]. These findings suggested the potential role of SLC7A5 in RA pathogenesis.

To investigate the function of overexpressed SLC7A5 in RA, we focused on the amino acid transportability of this molecule. We hypothesized that amino acid transport may modulate the FLS infiltration in RA. Our data shows that siRNA knockdown0 or antibody blocking of SLC7A5 suppressed the protein levels of MMP3 and MMP13 significantly. And these two proteinases are responsible to mediate the cleavage of aggrecan and collagen in damaged cartilage [24]. Raposo et al. found that using the amino acid transport inhibitor 2-(methylamino) isobutyric acid could attenuate the severity of arthritis in diseased animals [25], suggesting that the amino acids and their transporters might be the key factor in RA pathogenesis. As we mentioned before, some studies suggest the synovial infiltration and tumor cell-like behavior of FLS in RA. Likewise, many SLC7A5-related tumor studies have demonstrated a critical role of SLC7A5 in tumor migration and invasion. For example, Janpipatkul et al. showed that the downregulation of the SLC7A5 expression suppressed cholangiocarcinoma cell migration and invasion [26]. Further, SLC7A5 regulated by miR-126-3p exhibited a strong association with cellular migration and metastasis in thyroid cancer cells [27]. All these data support the participation of SLC7A5 in regulating cellular infiltration and invasion, and MMP expression in RA FLS. We observed that antibody blocking of SLC7A5 suppressed the MMP3 and MMP13 expression only at the protein level, and not affected their mRNA levels, suggesting that blocked SLC7A5 might contribute to modulate the amino acid-sensing mechanism. mTOR is a central nutrient sensor that signals a cell to grow and proliferate. One of the important functions of the mTOR complex (mTORC) is to maintain the available amino acid pool by regulating protein translation [28]. Dysregulation of the mTOR pathway leads to aberrant protein translation which manifests into various pathological states [29]. We showed that knocked down by siRNA, SLC7A5 could decrease the protein level of MMP3 and MMP13 via suppressing the phosphorylation of mTOR and P70S6K significantly. MMP3 and MMP13 expression was also decreased significantly when we used rapamycin to inhibit mTORC1 signaling. Ito et al. have shown that knockdown of Raptor (a component of mTORC1), reduced the MMP3 and MMP13 expression in nucleus pulposus cells of the human intervertebral disc treated with IL-1β [30]. Cejka et al. showed that inhibited mTORC1 via sirolimus or everolimus could reduce synovial osteoclast formation and protect against local bone erosions and cartilage loss [31]. Curcumin, another inhibitor of mTOR signaling, was also reported to alleviate rheumatoid arthritis-induced inflammation and synovial hyperplasia by reducing inflammatory mediators like IL-1β, TNF-α, MMP-1, and MMP-3 [32]. SLC7A5 mediates amino acid flux and activates mTORC1 signaling in tumors as well as immune cells [12, 22]. SLC7A5 knockout cancer cell lines showed decreased P70S6K phosphorylation and compromised cell proliferation [33]. Intracellular amino acids induce mTOR phosphorylation which activates its downstream target P70S6K [34]. P70S6K plays important roles in cell growth, proliferation, and differentiation by regulating cell cycle progression and ribosome biogenesis [35, 36]. It phosphorylates multiple components of the translational machinery and related regulators and increases translation by stimulating rRNA and tRNA synthesis [37]. It is reported that SLC7A5 together with SLC3A2 participates in transporting large neutral amino acids such as Phe, tyrosine, or Trp into the cell [38]. As shown in the present study, FLS cultured in a Trp-deficient medium exhibited a decreased expression of MMP13. In contrast, when FLS were cultured in the Trp supplemented medium, the expression of MMP13 was increased. At the same time, the supplemented Trp could activate the mTOR complex 1 signaling. Likewise, Kyn (a metabolite of Trp) was also able to activate mTOR signaling in T cells of SLE patients [39], suggesting that amino acids and their metabolites may also play important roles in regulating autoimmune response. Moreover, the upstream of mTOR signal, Akt activation, could also induce MMP3 and MMP13 expression in the microglia [40]. In the present study, we also found that SLC7A5 siRNA could cause increased IL-10 and TIMP1 and decreased PDGF-BB protein production in RA FLS supernatant. Previously, it was reported that IL-10 works as an anti-inflammatory cytokine, inhibits VEGF [41], and suppresses inflammatory response [42]. The increased Timp1 has been reported to ameliorate cartilage destruction in collagen-induced arthritis in rats [43]. All these findings are in accordance with our hypothesis that the amino acid transporter SLC7A5 takes part in cellular invasion and regulates protein levels of MMP3 and MMP13 via the mTOR signaling pathway in RA FLS. Downregulated or blocked SLC7A5 in FLS could serve as an anti-inflammatory molecule and a potential therapeutic target in arthritis.

## Conclusion

The present study highlights the important function of SLC7A5 in FLS from RA patients. IL-1β treatment of the cells causes higher expression of SLC7A5 through the NF-κB pathway. Blocking SLC7A5 activity inhibits MMP3 and MMP13 expression in FLS. Contrarily, overexpressed SLC7A5 enhances the protein production of MMP and MMP13 mediated by the mTOR-P70S6K-translation pathway. The findings provide new insights into the pathogenesis of RA and may pave the way for novel therapeutic strategies for the treatment of the disease.

## Supplementary information

**Additional file 1 **: **Fig. S1.** The SLC7A5 expression in RA and OA synovial tissues. **Fig. S2.** The differentially expressed cytokines and chemokines affected by SLC7A5 in RA FLS were analyzed by GO terms. (A). biological process, (B). molecular function, (C). cellular component. **Fig. S3.** The protein expression of SLC7A5 in FLS from RA patients incubated with different inhibitors and stimulated with IL-1β. **Fig. S4.** The protein expression of MMP3, MMP13 and SLC7A5 in FLS from RA patients blocked via SLC7A5 antibody (A) or siRNA (B). **Fig. S5.** Activation of mTOR-P70S6K signalling and downstream up-regulation of MMP3 and MMP13 expression by SLC7A5 over expressed in RA FLS. The mTOR-P70S6K signal activation after IL-1β treatment (A). The impact of SLC7A5 siRNA on the protein synthesis pathway (mTOR-P70S6K-4EBP1) activation in FLS (B). The inhibition of MMP3 and MMP13 expression by rapamycin (mTORC1 inhibitor) in RA FLS under IL-1β treatment (C). **Fig. S6**. mTOR-P70S6K-4EBP1 activation and downstream up-regulation of MMP13 by Trp in FLS from RA patients. **Table S1.** Primer. **Table S2.** Cytokine dot ELISA list. **Table S3.** Cytokine expression. **Table S4**. KEGG pathway analysis. **Table S5.** Primary antibodies.

## Data Availability

The data sets used and/or analyzed during the current study are available from the corresponding author on reasonable request.

## Abbreviations

CRP: C-reactive protein
ESR: Erythrocyte sedimentation rate
FLS: Fibroblast-like synoviocytes
Kyn: Kynurenine
MMPs: Matrix metalloproteinases
mTORC1: Mechanistic target of rapamycin complex 1
Phe: Phenylalanine
RA: Rheumatoid arthritis
RF: Rheumatoid factor
SLC7A5: Solute carrier family 7 member 5
Trp: Tryptophan
