# Supplementary material for: Intervening upregulated SLC7A5 could mitigate inflammatory mediator by mTOR-P70S6K signal in rheumatoid arthritis synoviocytes
Source: Arthritis Res Ther. 2020 Aug 31;22:200. doi: 10.1186/s13075-020-02296-8 (PMC7457370; doi:10.1186/s13075-020-02296-8)
Supplement: Supplementary file 1 — Additional file 1 : Fig. S1. The SLC7A5 expression in RA and OA synovial tissues. Fig. S2. The differentially expressed cytokines and chemokines affected by SLC7A5 in RA FLS were analyzed by GO terms. (A). biological process, (B). molecular function, (C). cellular component. Fig. S3. The protein expression of SLC7A5 in FLS from RA patients incubated with different inhibitors and stimulated with IL-1β. Fig. S4. The protein expression of MMP3, MMP13 and SLC7A5 in FLS from RA patients blocked via SLC7A5 antibody (A) or siRNA (B). Fig. S5. Activation of mTOR-P70S6K signalling and downstream up-regulation of MMP3 and MMP13 expression by SLC7A5 over expressed in RA FLS. The mTOR-P70S6K signal activation after IL-1β treatment (A). The impact of SLC7A5 siRNA on the protein synthesis pathway (mTOR-P70S6K-4EBP1) activation in FLS (B). The inhibition of MMP3 and MMP13 expression by rapamycin (mTORC1 inhibitor) in RA FLS under IL-1β treatment (C). Fig. S6. mTOR-P70S6K-4EBP1 activation and downstream up-regulation of MMP13 by Trp in FLS from RA patients. Table S1. Primer. Table S2. Cytokine dot ELISA list. Table S3. Cytokine expression. Table S4. KEGG pathway analysis. Table S5. Primary antibodies. [file 13075_2020_2296_MOESM1_ESM.docx]

Supplemental Fig.1


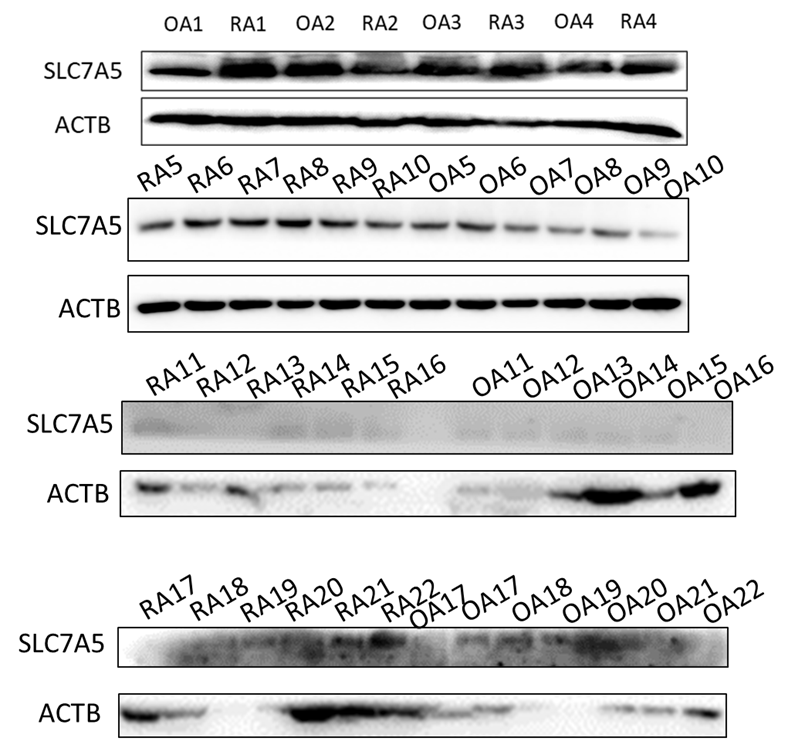


Fig. S1. The SLC7A5 expression in RA and OA synovial tissues.

Supplemental Fig.2


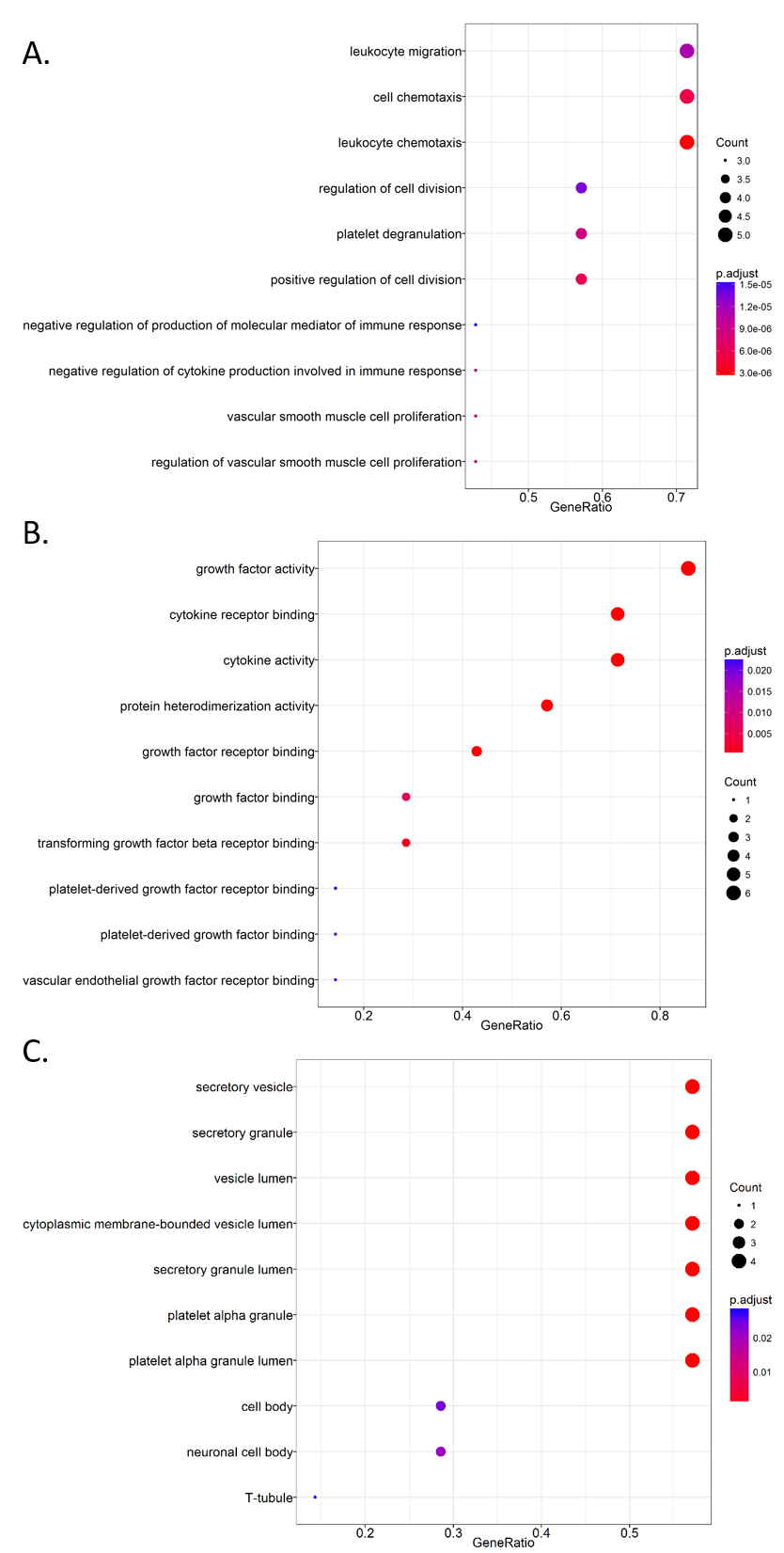


Fig. S2. The differentially expressed cytokines and chemokines affected by SLC7A5 in RA FLS were analyzed by GO terms. (A). biological process, (B). molecular function, (C). cellular component.

Supplemental Fig.3


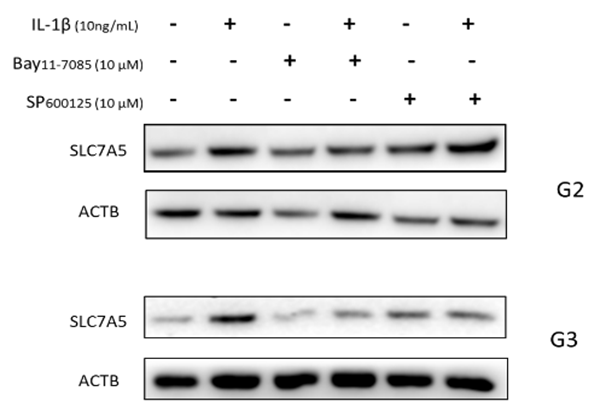


Fig. S3. The protein expression of SLC7A5 in FLS from RA patients incubated with different inhibitors and stimulated with IL-1β.

Supplemental Fig.4


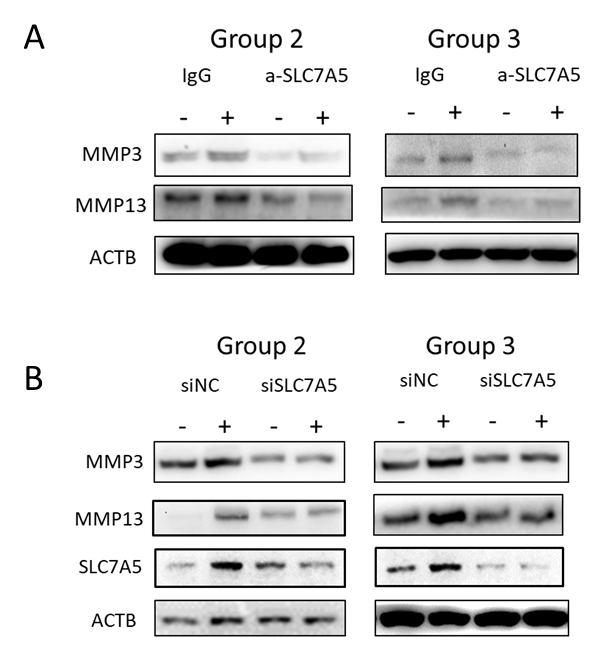


Fig. S4. The protein expression of MMP3, MMP13 and SLC7A5 in FLS from RA patients blocked via SLC7A5 antibody (A) or siRNA (B).

Supplemental Fig.5


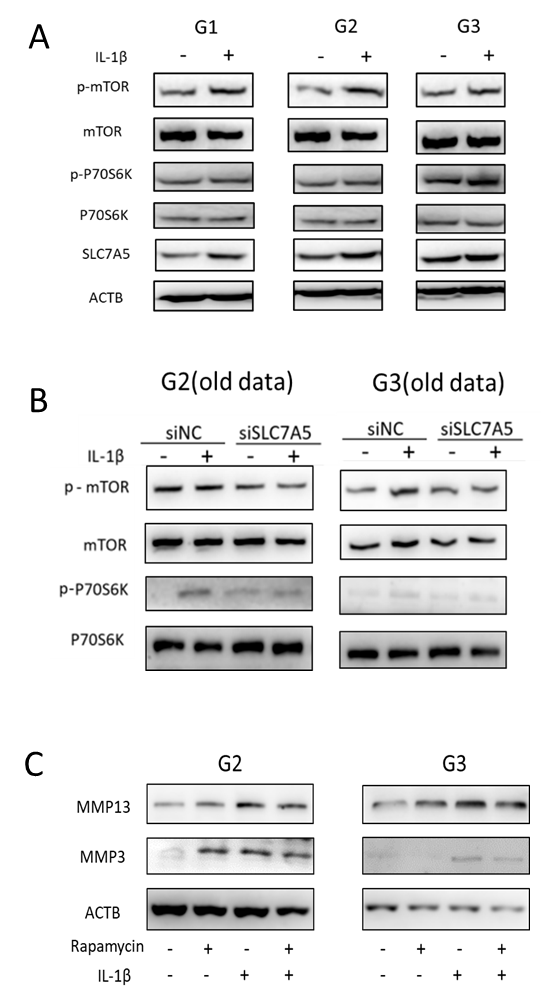


Fig. S5. Activation of mTOR-P70S6K signalling and downstream up-regulation of MMP3 and MMP13 expression by SLC7A5 over expressed in RA FLS. The mTOR-P70S6K signal activation after IL-1β treatment (A). The impact of SLC7A5 siRNA on the protein synthesis pathway (mTOR-P70S6K-4EBP1) activation in FLS (B). The inhibition of MMP3 and MMP13 expression by rapamycin (mTORC1 inhibitor) in RA FLS under IL-1β treatment (C).

Supplemental Fig.6


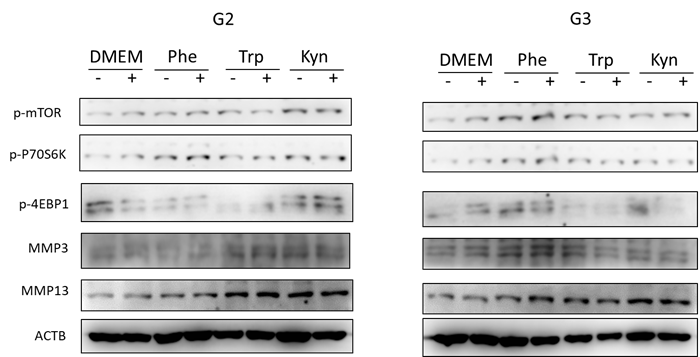


Fig. S6. mTOR-P70S6K-4EBP1 activation and downstream up-regulation of MMP13 by Trp in FLS from RA patients

**Supplemental material tables**

Table S1. Primer

| Gene symbol | | Sequence (5'-3') | PCR product size (bp) | Annealing temperature (℃) |
| --- | --- | --- | --- | --- |
| (GenBank accession number) | |  |  |  |
| *Slc7a5* | F | CCGTGAACTGCTACAGCGT | 113 | 60 |
| NM_003486.6 | R | CTTCCCGATCTGGACGAAGC |  |  |
| *Gapdh* | F | GGAGCGAGATCCCTCCAAAAT | 197 | 60 |
| NM_001256799 | R | GGCTGTTGTCATACTTCTCATGG |  |  |
| *Mmp3* | F | CTGGACTCCGACACTCTGGA | 79 | 60 |
| NM_002422.4 | R | CAGGAAAGGTTCTGAAGTGACC |  |  |
| *Mmp13* | F | AATATCTGAACTGGGTCTTCCAAAA | 102 | 60 |
| NM_002427.3 | R | CAGACCTGGTTTCCTGAGAACAG |  |  |

Table S2. Cytokine dot ELISA list


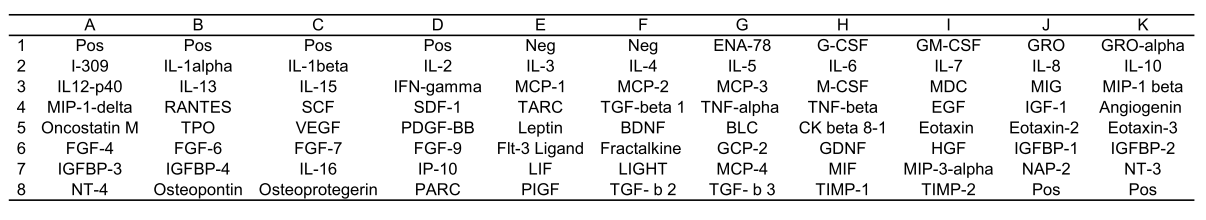


Table S3. Cytokine expression


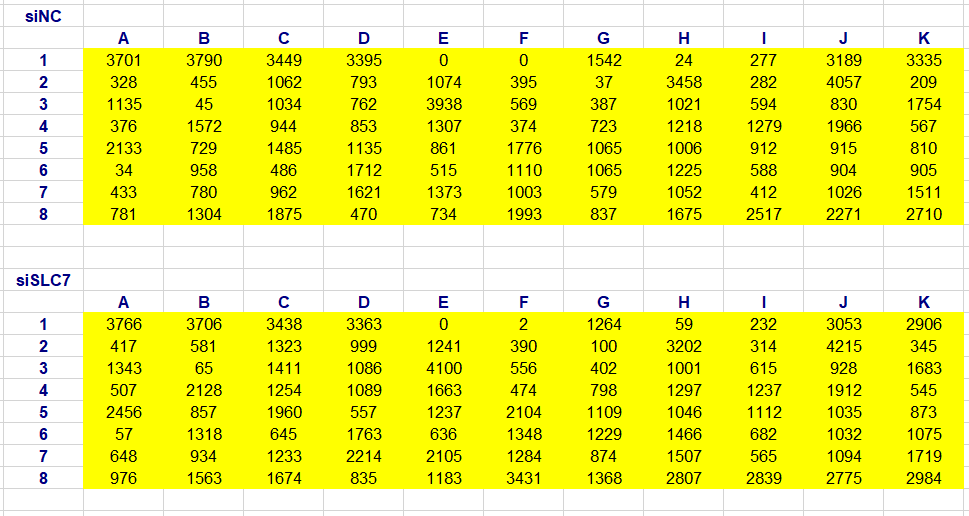


Table S4. KEGG pathway analysis


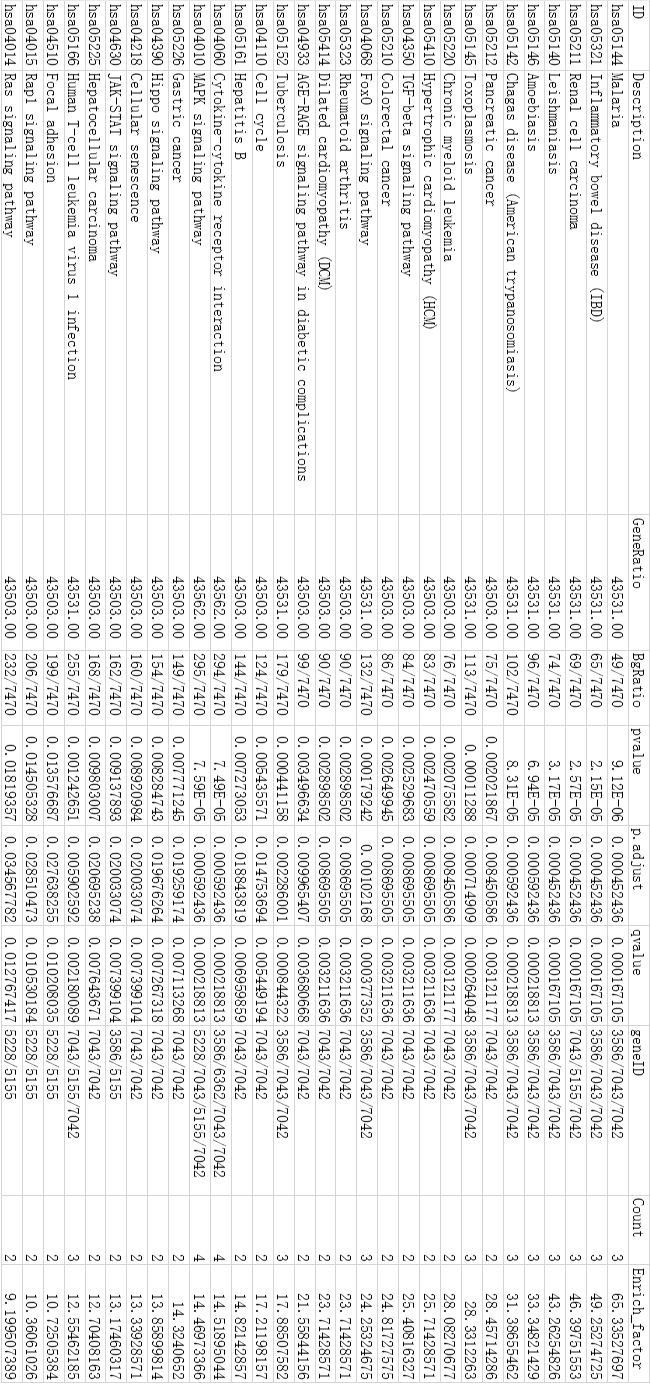


Table S5.Primary antibodies

| Primary antibody | Dilution ratio | Company |
| --- | --- | --- |
| rabbit anti-SLC7A5 | 1/200 | Boster, BA3479, China |
| rabbit anti-p-mTOR | 1/1000 | CST, #9862 kit, USA |
| rabbit anti-mTOR | 1/1000 | CST, #9862 kit, USA |
| rabbit anti-p-P70S6K | 1/1000 | CST, #9862 kit, USA |
| rabbit anti-P70S6K | 1/1000 | CST, #9862 kit, USA |
| rabbit anti-p-JNK1/2 | 1/2000 | CST, #4668, USA |
| rabbit anti-JNK1/2 | 1/1000 | CST, #9252, USA |
| rabbit anti-IκBα | 1/500 | Proteintech, 10268-1-AP, China |
| rabbit anti-MMP3 | 1/1000 | Abcam, ab53015, USA |
| rabbit anti-MMP13 | 1/1000 | Abcam, ab39012, USA |
| mouse anti-β-actin | 1/2000 | Earthox, E021020-03, USA |
